# Deep Reinforcement Learning-Based Intelligent Water Level Control: From Simulation to Embedded Implementation

**DOI:** 10.3390/s26010245

**Published:** 2025-12-31

**Authors:** Kevin Cusihuallpa-Huamanttupa, Erwin J. Sacoto-Cabrera, Roger Jesus Coaquira-Castillo, L. Walter Utrilla Mego, Julio Cesar Herrera-Levano, Yesenia Concha-Ramos, Edison Moreno-Cardenas

**Affiliations:** 1 TESLA Laboratory, Universidad Nacional de San Antonio Abad del Cusco (UNSAAC), Cusco 08003, Peru; 100343@unsaac.edu.pe (K.C.-H.); walter.mego@unsaac.edu.pe (L.W.U.M.); julio.herrera@unsaac.edu.pe (J.C.H.-L.); edison.moreno@unsaac.edu.pe (E.M.-C.); 2 Professional Academic School of Systems and Computer Engineering, Universidad Continental, Cusco 08000, Peru; 3 GIHP4C, Universidad Politécnica Salesiana, Cuenca 010102, Ecuador; esacoto@ups.edu.ec; 4 LIECAR Laboratory, Universidad Nacional de San Antonio Abad del Cusco (UNSAAC), Cusco 08003, Peru; roger.coaquira@unsaac.edu.pe

**Keywords:** deep reinforcement learning, water level control, DDPG algorithm, neural networks, Arduino Uno, real-time systems

## Abstract

This article presents the design, simulation, and real-time implementation of an intelligent water level control system using Deep Reinforcement Learning (DRL) with the Deep Deterministic Policy Gradient (DDPG) algorithm. The control policy was initially trained in a MATLAB-based simulation environment, where actor–critic neural networks were trained and optimized to ensure accurate and robust performance under dynamic and nonlinear conditions. The trained policy was subsequently deployed on a low-cost embedded platform (Arduino Uno), demonstrating its feasibility for real-time embedded applications. Experimental results confirm the controller’s ability to adapt to external disturbances. Quantitatively, the proposed controller achieved a steady-state error of less than 0.05 cm and an overshoot of 16% in the physical implementation, outperforming conventional proportional–integral–derivative (PID) control by 22% in tracking accuracy. The combination of the DDPG algorithm and low-cost hardware implementation demonstrates the feasibility of real-time deep learning-based control for intelligent water management. Furthermore, the proposed architecture is directly applicable to low-cost Internet of Things (IoT)-based water management systems, enabling autonomous and adaptive control in real-world hydraulic infrastructures. This proposal demonstrates its potential for smart agriculture, distributed sensor networks, and scalable and resource-efficient water systems. Finally, the main novelty of this work is the deployment of a DRL-based controller on a resource-constrained microcontroller, validated under real-world perturbations and sensor noise.

## 1. Introduction

Machine learning (ML) has revolutionized the design of Intelligent Systems (ISs), enabling data-driven solutions for tasks that previously relied on explicit modeling. Over the past decade, ML techniques have gained popularity in several fields, including robotics, healthcare, finance, and industrial control [1,2]. In particular, deep learning (DL), a subset of ML that leverages deep neural networks, has achieved cutting-edge results in computer vision, natural language processing, and control systems [3,4,5]. Within the DL paradigm, Deep Reinforcement Learning (DRL) stands out for its ability to address sequential decision-making problems where agents learn optimal behavior through interaction with an environment [6]. DRL has demonstrated remarkable success in high-complexity tasks such as video game playing [7], autonomous driving [8], and robotic manipulation [9]. Within this paradigm, the Deep Deterministic Policy Gradient (DDPG) algorithm is particularly well suited to environments with continuous actions due to its lower computational cost and stability in control tasks, compared to more recent alternatives such as SAC or TD3 [10,11].

In recent years, the use of artificial intelligence and embedded computing techniques has driven advances in intelligent monitoring and control in real-world applications. The integration of learning algorithms, fuzzy logic, and IoT infrastructure has enabled the regulation of environmental variables in hydroponic systems [12], the detection of faults in industrial refrigeration equipment [13], and the development of smart farming solutions based on sensors and images [14]. However, despite these advances, the implementation of DRL-based controllers in low-cost hardware remains limited due to inherent constraints such as memory capacity, processing power, PWM discretization, and noise tolerance of economic sensors.

Most studies applying DRL to process control remain exclusively in simulation [15,16], leaving open whether the learned policies can be reliably executed under real conditions. In particular, it is still unclear whether policies learned through DRL can be successfully implemented in resource-constrained microcontrollers, considering memory constraints, acoustic noise from ultrasonic sensors, relatively high sampling times, and discretization in actuators. The lack of physical validation has limited the adoption of DRL in level control systems, where classical solutions based on PID or fuzzy controllers predominate [12].

In this context, this paper addresses the gap directly through the design, training, and experimental validation of an intelligent controller based on DDPG for water level regulation. The proposed approach enables the evaluation of the agent’s performance under real-world disturbances and sensor noise, including HC-SR04 sensor noise, actuator latency, and hardware limitations inherent to the Arduino Uno microcontroller. This demonstrates the feasibility of deploying DRL controllers on low-cost embedded platforms, which is relevant for applications in IoT infrastructure, smart agriculture, and automatic control education.

The main contributions of this work are summarized below:

- A DDPG-based controller for water level regulation is implemented and experimentally validated on an Arduino Uno microcontroller, which has limited computational resources.
- A reproducible workflow is presented that covers system modeling, agent training in MATLAB, policy export, and real-time embedded execution.
- The performance of the controller under real-world conditions is analyzed, including sensory noise, PWM discretization, hydraulic variations, and actuator latency.
- The practical implications of using DRL in low-cost IoT and hydraulic systems are discussed, highlighting its potential for industrial and educational applications.

In summary, the novelty of this study is the proposal of a DRL-based water level control architecture that enables continuous learning and deployment on resource-constrained hardware. For this architecture, a DDPG agent is trained with optimized actor–critic networks, validating its performance both in simulation and in physical implementation under conditions of real disturbances and sensor noise. The results will allow us to determine the feasibility of implementing DRL policies in embedded systems with limited resources. This determination will open up new opportunities for the development of intelligent, adaptive, and low-cost control strategies in water management systems and technical learning environments.

### Related Works

In recent years, DRL has demonstrated its effectiveness as a tool for solving continuous control tasks in complex and nonlinear environments [17]. However, most of the reported advances have been validated primarily in simulations, where sensor noise, actuator delays, model mismatches, and hardware limitations do not exist. This has raised growing concern in the community about the transferability of DRL policies from simulation to real-world embedded platforms, especially in low-cost systems where memory, computing power, and sampling frequency are severely limited.

Among the various DRL algorithms, DDPG has gained prominence due to its effectiveness in continuous control problems and compatibility with actor–critic architectures [18]. Despite these advantages, several studies have highlighted their sensitivity to noise and hyperparameter tuning, which is particularly relevant in embedded implementations where filtering, sensor stability, and limited inference accuracy can significantly distort the learned policy [11]. Therefore, these aspects must be carefully evaluated when implementing DDPG on resource-constrained hardware.

Several authors have begun to address this gap. For example, in [15], the authors implemented DDPG on a Raspberry Pi-based platform to control a servo-driven robotic arm, demonstrating its feasibility with limited computational resources. Although this represents a step towards embedded DRL, Raspberry Pi boards offer significantly more memory and processing power than low-cost microcontrollers like Arduino or STM32, which limits the generalizability of their conclusions to highly constrained systems. Similarly, in [19], the authors proposed an improved DDPG algorithm for mapless path planning, introducing multi-step updates and dual-noise mechanisms to improve learning efficiency and navigation robustness. Real-world experiments confirmed improvements compared to standard DDPG. However, the implementation was based on hardware with capabilities substantially superior to those of typical IoT microcontrollers. Finally, the implementation reported in [20] on STM32 microcontrollers required extensive network pruning and quantification, highlighting the challenges posed by adapting DRL policies to very limited memory spaces.

In another context, DDPG was applied to the multivariable control of level and pH processes, outperforming classical controllers such as PID in terms of accuracy [21]. Despite these promising results, the study was conducted exclusively in simulation and omitted crucial aspects, such as sensor noise, actuator saturation, and the computational constraints of low-cost hardware. Similarly, the application of DDPG in HVAC systems [15] demonstrated reductions in energy consumption while maintaining thermal comfort. However, the experiments were based on simulations or high-level computer platforms, leaving unresolved questions about the agent’s stability in real environmental disturbances and limited sampling rates. Likewise, in intelligent transportation, the authors in [16] applied DDPG to lane change maneuvers and adaptive cruise control, achieving exceptional performance in vehicle simulators and demonstrating strong generalization in the face of modeling errors. However, these algorithms were not validated on integrated automotive controllers or tested under real sensory noise or latency, reinforcing the trend that most DDPG applications remain limited to simulation environments.

Based on the above, most DDPG-related studies have demonstrated their effectiveness in controlled virtual environments. However, few have taken the step towards experimental validation in hardware with strict resource constraints or low-cost, noisy sensors. Unlike these studies, the present research proposes a comprehensive architecture that encompasses hydraulic modeling, DDPG training in MATLAB, integrated implementation on an Arduino Uno, and real-time evaluation under realistic disturbances. Furthermore, this work not only demonstrates the feasibility of implementing DRL controllers on low-cost embedded hardware but also provides quantitative evidence of their performance in the presence of sensor noise, actuator discretization, and hydraulic variability. Furthermore, the simplicity and reproducibility of the proposed system make it a valuable platform for education and research in intelligent control, addressing an unexplored gap in previous DRL studies that have focused primarily on simulation or high-capacity hardware.

On the other hand, in most previous studies, tank level controllers have only been implemented in simulation environments or using conventional strategies, such as PID or fuzzy logic. Although these strategies work well under ideal conditions, they rely heavily on manual tuning and the system model. Few studies have reported physical validations of intelligent controllers based on deep reinforcement learning. However, this work explicitly addresses this research gap by designing, training, and implementing a DDPG controller on a low-cost microcontroller, demonstrating its feasibility and stability under real-world conditions and offering an autonomous and adaptable alternative to classical control methods.

The article is structured as follows. Section 2 describes the proposed model and specifies the technical characteristics. Section 3 presents the main results obtained after training and implementing the controller based on the DDPG algorithm. Section 4 draws the main conclusions. Finally, in Section 5 presents the future research issues.

## 2. Materials and Methods

**Figure 1:**
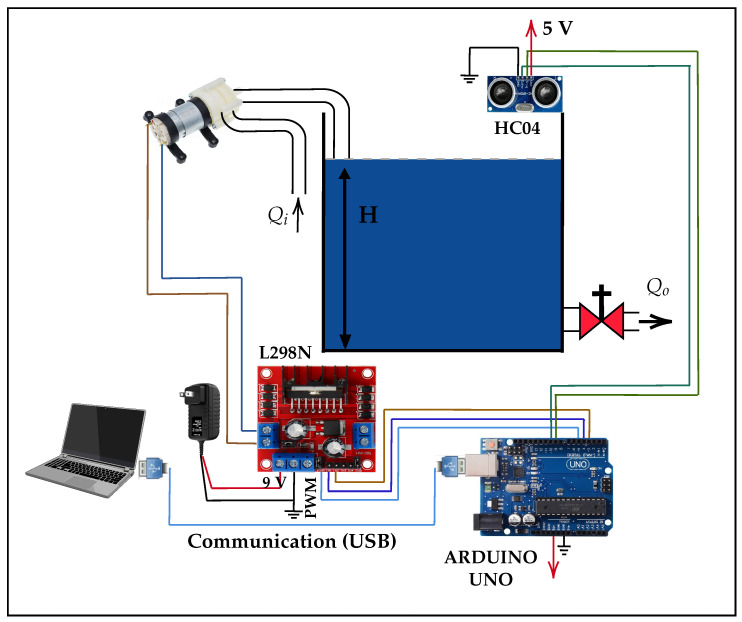
Diagram of the physical system implemented for water level control.

In this section, we propose a system model consisting of a plant that automatically controls the water level in a tank using DLR. The implementation of the system, as shown in Figure 1, consists of an acrylic tank with a useful height of 20 cm, an HC-SR04 ultrasonic sensor (Manufacturer: Generic manufacturer; Shenzhen, China) located at the top of the tank to measure the water level, and a 12 V motor pump that acts as the main actuator responsible for supplying water to the system. An outlet valve with a fixed opening has also been installed to simulate constant demand. The motor pump is controlled by an L298N power controller (Manufacturer: STMicroelectronics; Geneva, Switzerland), which is governed by an Arduino Uno microcontroller (Manufacturer: Generic manufacturer; Shenzhen, China) that executes the learned control policy. In addition, an external 9 V power supply is used to power the motor pump, while the Arduino is powered via a USB connection. All components were mounted on a wooden base, and the electronic elements were protected with a plastic casing, ensuring the system’s integrity against splashes or adverse environmental conditions.

The implementation of the system model is structured in three stages. The first stage is the mathematical modeling of the water level control system. The second stage is the design and simulation of the reinforcement learning controller. The third stage is the implementation of the system model. These stages are described in Section 2.1, Section 2.2 and Section 2.3. Table 1 shows the general notation used in this article.

### 2.1. Mathematical Modeling of the Water Level Control System

This section describes the process followed to define and adjust the mathematical model that adequately describes the dynamic behavior of the water level system. The objective of this model is to develop an efficient system controller. The model also serves as the basis for both the simulation of the control agent and the subsequent performance analysis. In addition, the modeling process begins with the theoretical formulation of the system using a mathematical model, followed by the experimental estimation of the parameters and, finally, the validation of the model with real data.

#### 2.1.1. Mathematical Model

**Figure 2:**
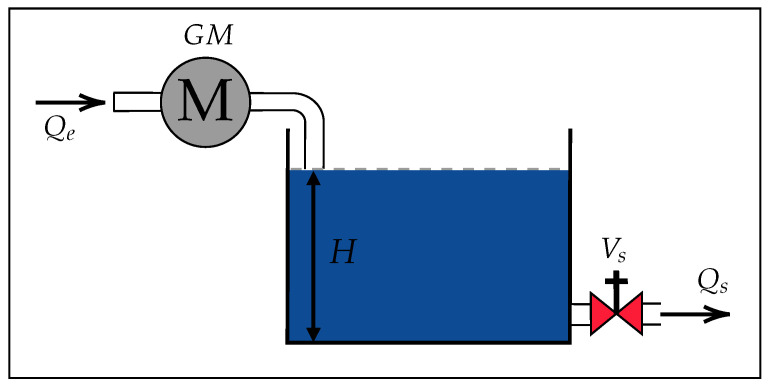
Water tank model.

The mathematical model of a tank’s water level control system is based on two main flows: the inflow, $Q_{e}$, and the outflow, $Q_{s}$, as shown in Figure 2. $Q_{e}$ and $Q_{s}$ are described using fluid dynamics principles [22]. $Q_{e}$ is modeled in Equation (1), while Qs is modeled in Equation (2). Both flow models are based on Torricelli’s Equation [22]. The validity of these equations is based on the fact that the flow regime observed during the emptying tests remained laminar, with estimated Reynolds numbers (Re < 2000) for heights below 20 cm and an effective outlet diameter of approximately 10 mm. Furthermore, according to classical fluid mechanics criteria [23], these physical characteristics place the system within the laminar range, where fluid behavior is stable and non-turbulent. Possible losses due to friction or turbulent effects are incorporated into the discharge coefficient represented by the parameter $k_{2}$.

(1) $$Q_{e}=k_{1}u_{1}$$

(2) $$Q_{s}=k_{2}a_{2}\sqrt{2gH}$$

where $k_{1}$ and $k_{2}$ are the inlet and outlet adjustment coefficients, $a_{2}$ is the degree of opening of the outlet valve, *g* is the acceleration due to gravity, *H* is the height of the water in the tank, and $u_{1}$ is the average voltage of the inlet motor. On the other hand, the variation in *H* defined in Equation (3) is modeled through a flow balance in the tank.

(3) $$A\frac{dH}{dt}=Q_{e}-Q_{s}$$

where *A* is the cross-sectional area of the tank, which is assumed to be constant, and represents the surface area over which *H* varies. Furthermore, by substituting Equations (2) and (1) into (3), the following Equation is obtained.

(4) $$A\frac{dH}{dt}=k_{1}u_{1}-k_{2}a_{2}\sqrt{2gH}$$

where $a_{2}$ is expressed as a dimensionless value in the range [0, 1]; this model is used in the following sections to estimate the parameters $k_{1}$ and $k_{2}$ from experimental data. The model is also used to validate itself by comparing it with the system’s actual response results.

#### 2.1.2. Experimental Estimation of Parameters

The values of the coefficients $k_{1}$ and $k_{2}$ are determined through experimental tests under controlled conditions. In these tests, the inlet and outlet flows of the tank were measured indirectly from the volume changes observed during known time intervals.

*Estimation of $k_{1}$.* The first set of experimental tests consists of filling the tank with the outlet valve completely closed, while keeping the motor pump activated at a constant average voltage, $u_{1}$. Under these conditions, several repetitions are performed to record the increase in *H* as a function of time. From Equation (1), $k_{1}$ is estimated using Equation (5).

(5) $$k_{1}=\frac{Q_{e}}{u_{1}}$$

**Figure 3:**
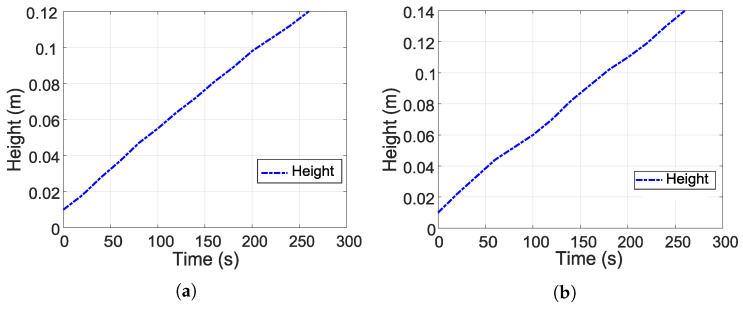
Experimental tests for estimation of parameter $k_{1}$. (**a**) Test 1: tank emptying, valve 50% open. (**b**) Test 2: tank emptying, valve 100% open.

Figure 3 shows the experimental tests for estimating $k_{1}$. Figure 3a,b show the experimental evolution of the water level during tank filling with the outlet valve closed. In Figure 3a, a 70% Pulse Width Modulation (PWM) signal is applied, while in Figure 3b, an 80% PWM signal is used. 100% PWM is equivalent to a signal of approximately 9.5 V. Likewise, an almost linear increase in *H* as a function of time can be observed in both figures, indicating that the inflow remained practically constant during the experiment. This behavior allows $Q_{e}$ to be calculated by estimating the slope of each curve.

The increase in the PWM value between the two tests produced a steeper slope, as shown in Figure 3b, which indicates a higher $Q_{e}$. In addition, these two representative curves were selected to show the filling process since the variability between the different tests was minimal. Finally, this information is used to estimate $k_{1}$ using Equation (5).

*Estimation of $k_{2}$.* The second set of experimental tests starts at *H* with the motor pump turned off and the outlet valve open at 60% of its total opening, i.e., $a_{2}=0.6$. In addition, the water is allowed to flow freely, and the evolution of the level is recorded during the emptying. The procedure is repeated in three tests. Based on Equation (2), $k_{2}$ is estimated using Equation (6).

(6) $$k_{2}=\frac{Q_{s}}{a_{2}\sqrt{2gH}}$$

**Figure 4:**
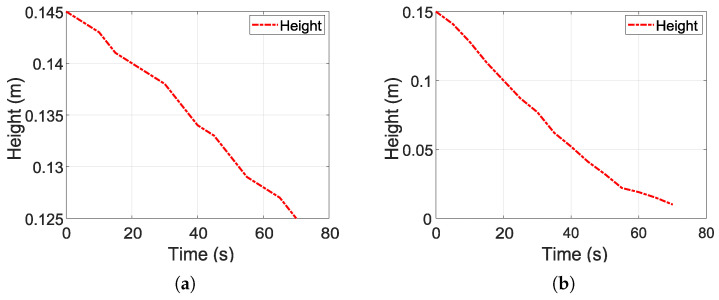
Experimental tests for estimation of parameter $k_{2}$. (**a**) Test 1: tank emptying, valve 50% open. (**b**) Test 2: tank emptying, valve 100% open.

On the other hand, Figure 4 shows the experimental tests for estimating $k_{2}$. Figure 4a,b show the emptying of the tank from an initial average height of 15 cm. In both figures, an approximately linear behavior in the decrease in *H* with time is observed, facilitating the estimation of $k_{2}$. On the other hand, in the first test (Figure 4a), the outlet valve was opened to 50% of its total opening. Based on this condition, the emptying was progressive, reaching a height of approximately 12.5 cm in 70 s. On the other hand, in the second test (Figure 4b), the valve is opened completely (100%), which generates a much faster drop in *H*, reaching almost complete emptying before 70 s. This difference between the two tests shows the direct effect of the outlet area on the emptying speed.

The representation of the *H* curves in both figures is used as part of the dataset to estimate $k_{2}$ using a nonlinear regression fit to the experimental data on tank filling and emptying. The joint estimation of $k_{1}$ and $k_{2}$ is performed using multiple representative tests due to their consistent and reproducible behavior. Although exact confidence intervals were not calculated, the values were obtained with low residual error, indicating that the regression provides a consistent and reliable estimate of the model parameters.

Based on the data obtained, the most representative values for $k_{1}$ and $k_{2}$ are determined, as shown in Table 2.

#### 2.1.3. Model Validation

**Figure 5:**
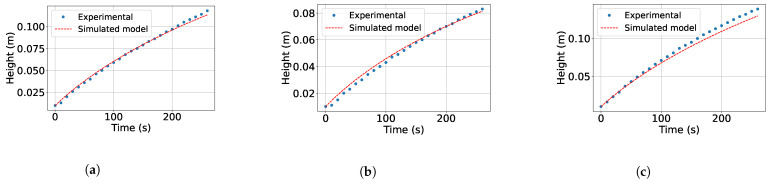
Experimental results under different conditions. (**a**) PWM =100 and $a_{2}=60$%. (**b**) PWM =70 and $a_{2}=50$%. (**c**) PWM =100 and $a_{2}=40$%.

The proposed mathematical model is validated by estimating $k_{1}$ and $k_{2}$, considering the water inlet controlled by the motor pump and the outlet regulated by the partially open valve. Thus, three experimental tests were performed using different combinations of PWM signal and valve $a_{2}$ opening percentage. Figure 5 compares the experimental data and the simulation results obtained using the proposed model. Figure 5a–c show a high degree of agreement between the two curves, which validates the model’s accuracy under varying operating conditions.

In Figure 5, we can observe that the evolution of *H* estimated by the model reproduces the trend observed experimentally with high precision. This evolution confirms that both the structure of the model and the parameters obtained adequately represent the dynamics of the physical system, which makes it a reliable tool for the analysis and design of automatic control strategies.

### 2.2. Design and Simulation of the DRL Controller

**Figure 6:**
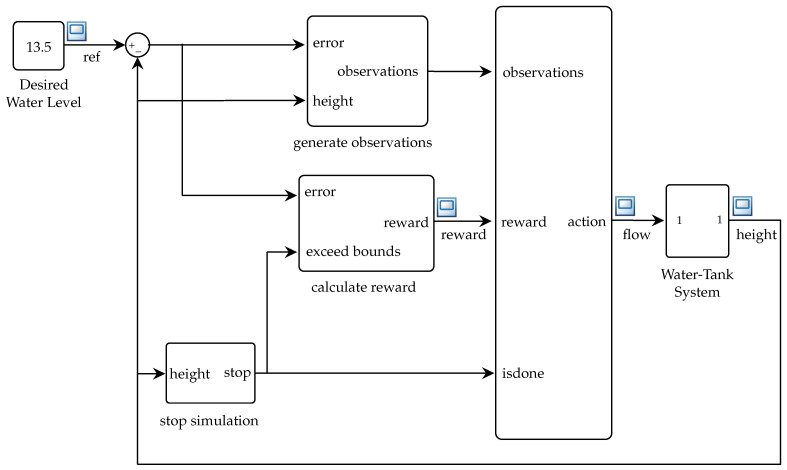
Diagram of the control structure based on DRL.

The DRL controller simulation is based on the structure implemented in Simulink, as shown in Figure 6. The design consists of four main aspects: DRL approach, DDPG algorithm, observation, action, and reward spaces of the agent, neural network configuration (actor network and critic network), and finally, hyperparameters and training. The DDPG algorithm was selected over other deep reinforcement learning alternatives, such as TD3 or SAC, due to its lower computational complexity and deterministic policy characteristics, which are advantageous for its execution in embedded systems. In particular, DDPG requires fewer stochastic updates and converges more quickly in continuous but low-dimensional action spaces, such as those found in flow regulation in hydraulic systems.

#### 2.2.1. DRL Approach

The DRL approach [24] is used in the design of the implemented structure because it offers an adaptive solution for *H* control, as it does not require an explicit mathematical model of the system, which is useful in the face of non-linear dynamics, delays, or uncertainties in the process. Furthermore, through this approach, a control agent is trained to observe key system variables, such as the water level and its rate of change, and take appropriate continuous actions; i.e., it adjusts the inflow rate to maintain the level within a desired range. The agent also learns optimal control policies by maximizing a reward signal to reflect the system’s performance, achieving efficient performance under disturbed or changing conditions.

#### 2.2.2. DDPG Algorithm

The DDPG algorithm is used in the design of the implemented structure, as a DRL-based control strategy, due to its ability to operate in environments with continuous action spaces [24]. This approach is suitable for regulating *H*, where the control action (flow adjustment) requires continuous values.

The DDPG agent is composed of an *actor–critic* architecture, where the *actor* network generates deterministic actions at $a_{t}=\mu \left(s_{t}|\theta^{\mu }\right)$ [11,25], and the *critic* network evaluates these actions by estimating the value function Q(s,a) [24]. During the training phase, mechanisms are implemented to stabilize learning, such as the use of *target* networks for both main networks and a replay buffer, which allows learning from historical samples. In addition, exploratory noise, specifically of the Ornstein–Uhlenbeck type, is incorporated to encourage exploration of the action space [11,25].

**Figure 7:**
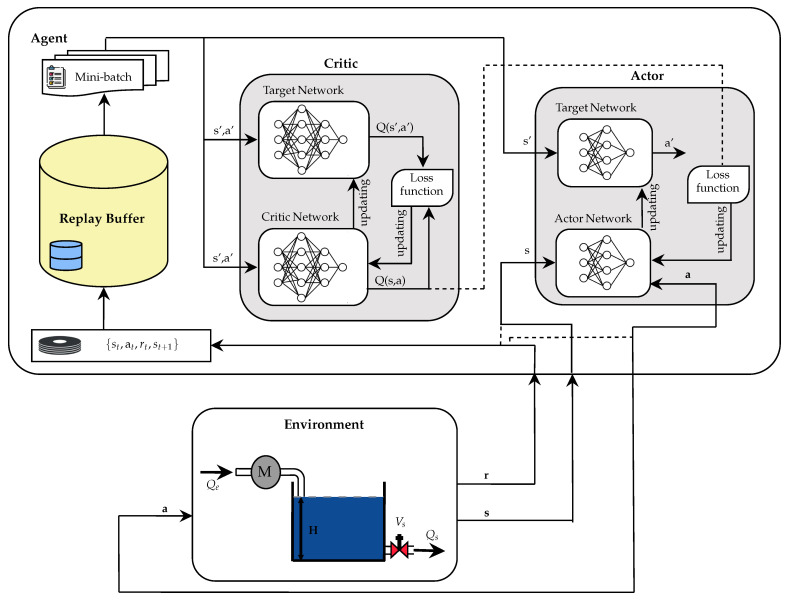
DDPG algorithm structure.

Figure 7 shows the training procedure, which follows the following steps.

- First, the *actor* and *critic* networks are configured with random weights, their copies are created as *objective* networks, the *replay buffer* is initialized, and the environment is defined.
- Then, the agent gets the initial state $s_{0}$ and selects an action $a_{t}$ with added scan. After executing the action in the environment, the reward $r_{t}$, the new state $s_{t+1}$, and a completion indicator (*done*) are obtained.
- The transition $\left(s_{t},a_{t},r_{t},s_{t+1},done\right)$ is buffered, from where a minibatch is extracted to update the *critic* network by minimizing the error between the current output $Q(s_{i},a_{i}|\theta^{Q})$ and the target value. This process is performed using Equation (7).
  (7) $$y_{i}=r_{i}+\gamma Q^{\prime }\left(s_{i+1},\mu^{\prime }\left(s_{i+1}|\theta^{\mu^{\prime }}\right)|\theta^{Q^{\prime }}\right)$$
- Subsequently, the *actor* network is updated by maximizing the *Q* value estimated by Equation (8).
- Finally, a smooth update of the target networks is performed by exponential interpolation using Equation (9).

(8) $$\nabla_{\theta^{\mu }}J\approx \frac{1}{N}\sum \nabla_{a}Q\left(s,a|\theta^{Q}\right){|}_{a=\mu (s)}\nabla_{\theta^{\mu }}\mu \left(s|\theta^{\mu }\right)$$

(9) $$\theta^{Q^{\prime }}\leftarrow \tau \theta^{Q}+\left(1-\tau \right)\theta^{Q^{\prime }},\;\theta^{\mu^{\prime }}\leftarrow \tau \theta^{\mu }+\left(1-\tau \right)\theta^{\mu^{\prime }}$$

This cycle is repeated until the defined number of episodes is completed or until the convergence of the agent is reached.

#### 2.2.3. Observation, Action, and Reward Spaces of the Agent

The DDPG agent interacts with the environment through a defined set of inputs (observations), actions, and a reward function [24], which guides the learning process towards a desired behavior.

- **Observation space.** The agent receives as input an observation vector that describes the system state at time instant *t*, as shown in Equation (10):
  (10) $$o=\left[\begin{array}{c} \int_{0}^{t}e\left(\tau \right)\;d\tau \\ e(t)\\ h(t) \end{array}\right],$$
  where $\int_{0}^{t}e\left(\tau \right)\;d\tau$ represents the accumulated error integral, which is useful for assessing historical behavior; $e\left(t\right)=h_{\mathrm{target}}-h\left(t\right)$ denotes the instantaneous error; and h(t) is the current water level in the tank as a function of time. This combination provides the agent with a rich representation of the state, encompassing history, trend, and the current condition of the system [26].
- **Action space.** The agent issues a continuous action a(t) at each time step, as defined in Equation (11):
  (11) $$a\left(t\right)=q_{\mathrm{in}}\left(t\right),$$
  where $q_{\mathrm{in}}\left(t\right)$ is the inflow rate to the tank, which is directly related to the power applied to the motor pump. This action is implemented using a PWM signal that regulates the motor speed and water flow.
- **Reward function.** The function defined in Equation (12) provides feedback that guides the agent’s learning. Its design encourages accurate tracking of the desired water level and avoids hazardous conditions [24].
  (12) $$R\left(t\right)=\left\{\begin{array}{cc} +10, & \mathrm{si}\;|e(t)|<0.1\;\mathrm{cm},\\ -1, & \mathrm{si}\;|e(t)|\geq 0.1\;\mathrm{cm},\\ -100, & \mathrm{si}\;h(t)<0\;\mathrm{cm}\;o\;h(t)>16\;\mathrm{cm}. \end{array}\right.$$
  This structure rewards precision, i.e., |e(t)|<0.1cm, penalizes larger deviations, and severely punishes operation outside the safety limits. In this way, the agent learns to regulate the water level accurately and safely over time. To improve robustness against measurement noise, a moving average filter with a window of three samples was applied to the water level readings during simulation. This pre-filtering reduced transient oscillations in the error and promoted faster convergence of the reward during training.

#### 2.2.4. Actor and Critic Networks

The actor network, which learns a deterministic policy $\mu (s|\theta^{\mu })$ to map observations to continuous actions (in this case, the inflow rate to the tank); and the critic network, which estimates the action-value function $Q(s,a|\theta^{Q})$, representing the expected return of taking action *a* in a given state *s* [11,24,25]. The actor network takes three observations as input: the integral of the error, the current error, and the water level in the tank. The output of the actor network is a continuous action that regulates the voltage applied to the motor pump. On the other hand, the critic network evaluates the action suggested by the actor and the system state. It estimates its value using an architecture that merges state and action information in separate processing streams.

**Figure 8:**
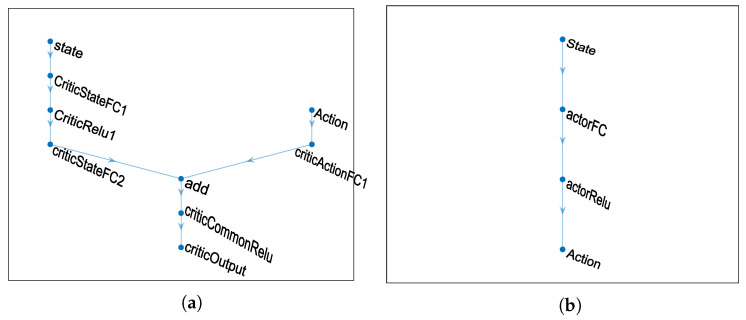
General architecture of neural networks generated with MATLAB. (**a**) Critical network. (**b**) Actor network.

Figure 8 illustrates the general architecture of the neural networks generated using MATLAB: the critic network (Figure 8a) and the actor network (Figure 8b). This representation provides a structural overview of how the layers in each network are interconnected.

Table 3 details the critical network architecture, while Table 4 details the actor network architecture. Both tables detail the internal architecture of both networks, specifying the number of neurons, the activation functions used, and the modular division of the critic network into the *State*, *Action*, and *Common* subnetworks, which are merged in the final stage. This information is essential for reproducing the proposed agent design.

#### 2.2.5. Hyperparameters and Training

The hyperparameters were tuned through an iterative empirical process. This procedure evaluated the stability of the average reward and the convergence of the agent, following a common practice in DDPG due to its sensitivity to initial settings. Table 5 summarizes the main hyperparameters configured for the DDPG agent. The final values were obtained after testing variations in parameters such as learning rates, minibatch size, and exploratory noise to evaluate their effect on training stability and overall performance.

A discount rate of γ=1.0 was used, along with an experience buffer of $10^{6}$ transitions and training mini-batches of 64 samples. A soft update technique was applied to the target networks with a value of τ=0.001 to stabilize learning. Exploration during training was encouraged by adding *Ornstein–Uhlenbeck* noise, which introduces temporal correlation and is suitable for environments with continuous action spaces [11,25]. This work set an initial variance of 0.3, with a gradual decay of $10^{-5}$.

Training was carried out in a simulated environment over 1000 episodes, each lasting 200 steps, to learn a policy capable of maintaining the water level at the reference value under varying initial conditions.

All experiments were performed using a fixed random seed (42) to ensure reproducibility of results. The source code and training parameters are publicly available and are mentioned in the Data Availability Statement section at the end of this article.

**Figure 9:**
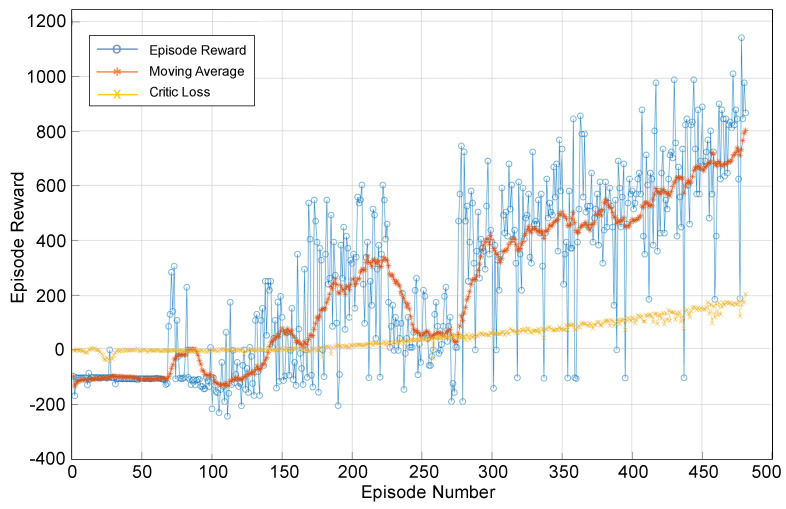
DDPG agent training process. Episode Reward For Water Tank with DPPG Agent.

Figure 9 shows the training process of the DDPG agent. The blue dotted line represents the reward obtained in each episode, which exhibits high variability due to exploration during learning. The orange line shows the reward’s moving average, highlighting the agent’s general performance trend. The yellow line represents the baseline performance, corresponding to an initial or random policy. Finally, a progressive improvement in the agent’s performance can be observed as the number of episodes increases.

### 2.3. Hardware Implementation of the DDPG Driver

This section describes the physical implementation process of the DDPG controller on an embedded platform based on Arduino. For this purpose, the configuration of the hardware used (embedded system) and how the control algorithm is integrated are described.

#### 2.3.1. Embedded System Configuration

The controller architecture was simplified to enable its implementation in embedded hardware with limited resources. In this version, only the actor network was used, consisting of a hidden layer of four neurons with ReLU activation, which significantly reduces computational demand. The weights and biases obtained during training were manually encoded as floating-point arrays in the Arduino program for forward pass calculation using basic arithmetic operations. This compact structure maintains the accuracy required for water level control, ensuring real-time execution with minimal memory usage.

The physical implementation of the control system is carried out using an embedded platform based on the Arduino Uno microcontroller, which is responsible for executing the control algorithm, acquiring sensor data, and generating the PWM control signal. The HC-SR04 ultrasonic sensor is used to measure the water level in the tank. This sensor, located at the top of the tank, provides real-time water level measurements using ultrasonic pulses. The control signal is applied to a 9 V motor pump responsible for filling the tank, whose speed was modulated through a PWM signal generated by the Arduino.

On the other hand, an L298N driver module is used for power management, which amplifies the PWM signal to control the motor pump efficiently. The system is powered by an external 9 V source for the pump, while the Arduino is powered via the USB connection. The configuration is designed to ensure stable and safe operation, with a sampling frequency of 4 s, thus ensuring an adequate response to changes in the water level. The schematic diagram of the embedded assembly, including the interconnections between the main components, was previously shown in Figure 1.

#### 2.3.2. Integration of the DDPG Controller into the Embedded Environment

The implementation of the controller based on the DDPG algorithm in an embedded environment is carried out by exporting the learned policy (actor model) and coding it in C++ on an Arduino Uno microcontroller for execution [27]. Likewise, a compact neural network with a single hidden layer with four neurons with ReLU activation function and an output neuron with ReLU activation is chosen, whose design was obtained from the training process in MATLAB [28] due to the limited resources of the Arduino.

The system operates at a sampling frequency of 4 s, using the TimerOne library [29] to generate periodic interrupts. In each sampling cycle, the distance is acquired using an HCSR04 ultrasonic sensor [30], which is converted to water level using a linear calibration defined by Equation (13). Likewise, the calibration of the HC-SR04 sensor showed good linearity, with a root-mean-square error (RMSE) of approximately 0.29 cm and a standard deviation of 0.26 cm, values consistent with its nominal accuracy of ±0.3 cm. Therefore, although a threshold of ±0.1 cm was used as a theoretical reference in evaluating the controller, the effective detection resolution of the system is ±0.3 cm. This practical limit was taken into account when interpreting the stability and small oscillations observed in the experimental water level measurements.

(13) H=−0.967·x+18.568

where *x* is the distance in centimeters, the error concerning the target level, setpoint, is calculated, and its integral is accumulated. These three values, error, integral of error, and current height, form the input vector of the neural network. This neural network, already trained with the DDPG algorithm, estimates the optimal action through flow rate, which is converted into a PWM signal. This PWM signal is scaled by a gain and restricted to the allowed range to control the speed of the motor pump. Likewise, the neural network implemented in the Arduino Uno corresponds only to the actor model, with reduced architecture (3–4–1), which allows it to adjust to the limitations of the microcontroller. The weights and biases were stored as float arrays in the program’s Flash memory, without the need for additional quantization. The compiled sketch used 8.838 bytes (27%) of program space and 385 bytes (18%) of dynamic memory, confirming that the implementation occupies less than a third of the available resources and is fully compatible with real-time execution.

The entire reading, processing, and control process is executed periodically within the control loop. Algorithm 1 presents the pseudocode that the DDPG controller executes in real time for the operation of the embedded system.
**Algorithm 1** Water level control using a DDPG-Trained neural networkINITIALIZATION()     Initialize neural network parameters (Actor trained via DDPG) $b_{i}^{\left(1\right)},w_{ji}^{\left(1\right)},b^{\left(2\right)},w_{i}^{\left(2\right)}$     Initialize global variables     Configure hardware: ultrasonic sensor, PWM output, LED indicators     Set sampling interval T=4 s     Initialize: $e_{t}\leftarrow 0$, i_error←0 CONTROL_LOOP()**while** System is running **do**             Read distance from ultrasonic sensor             nivel←a·distancia+b             $e_{t}\leftarrow setpoint-nivel$             $i\_error\leftarrow i\_error+e_{t}\cdot T$             limit $e_{t}$, i_error whitin a safe range             $x\leftarrow [i\_error,\;e_{t},\;nivel]$             u← NeuralNetwork(*x*)             PWM_output← Gain · *u*             Apply PWM and activate fixed-direction pump             Wait *T* seconds**end while**

The neural network implemented in the embedded environment corresponds to the actor model trained using the DDPG algorithm. This neural network consists of an input layer with three nodes: error integral, instantaneous error, and water level; a hidden layer with four neurons using the ReLU activation function; and an output layer with ReLU activation. The weights and biases were obtained from the training environment in MATLAB and transferred to the C++ code for Arduino.

On the other hand, the evaluation procedure for this neural network is detailed in Algorithm 2. During execution on the microcontroller, the input vector *x* is formed in each control cycle, propagating forward in the network. First, the output of each hidden neuron is calculated by applying a linear combination of the inputs plus the bias, followed by the ReLU activation. Then, these outputs are linearly combined with the weights of the output layer and their respective bias, and finally, the ReLU activation, returning the action *u*. This value determines the PWM signal that activates the motor pump.
**Algorithm 2** Actor neural network evaluation**Input:** $x=[x_{1},x_{2},x_{3}]$Initialize h=[0,0,0,0]**for** i=1 to 4 **do**      $h_{i}\leftarrow b_{i}^{\left(1\right)}$      **for** j=1 to 3 **do**            $h_{i}\leftarrow h_{i}+w_{ji}^{\left(1\right)}\cdot x_{j}$      **end for**      $h_{i}\leftarrow \mathrm{ReLU}\left(h_{i}\right)$**end for**$y\leftarrow b^{\left(2\right)}$**for** i=1 to 4 **do**      $y\leftarrow y+w_{i}^{\left(2\right)}\cdot h_{i}$**end for**u←ReLU(y)**Return:** *u*

#### 2.3.3. Control Algorithm

The simulation utilized a sampling time of 1 s to expedite training, whereas the physical implementation employed a 4-s sampling time due to limitations in microcontroller acquisition and processing. Since the hydraulic dynamics have time constants of several seconds, this difference does not affect stability or performance, maintaining a consistent response between simulation and experiment.

The embedded control algorithm is executed in the microcontroller and is repeated cyclically every T=4 s, following a sequence of key steps.

- First, the system starts and reads the distance from the HC-SR04 ultrasonic sensor. This distance is converted into water level using a previously calibrated linear relationship.
- Next, the error is calculated as the difference between the desired value (setpoint) and the current level, and the error integral is updated, accumulating the history of the system’s behavior.
- Once these variables are obtained, the input vector is constructed and fed into the trained neural network (actor model). This network evaluates the system’s state and generates a continuous control action, which is scaled and transformed into a PWM signal that regulates the speed of the motor pump.
- Finally, the PWM signal is applied to the actuator through the corresponding control pin, and the system waits for the next sampling interval to repeat the process.

This closed control loop runs indefinitely while the system is active, ensuring continuous regulation of the water level in the tank. The real-time performance of the controller was evaluated during execution on the Arduino Uno microcontroller. The control cycle is executed by timer interrupts every 4 s, which is suitable for the slow dynamics of the hydraulic system (time constant approx. 10 s). The processing time of the actor’s neural network, which includes three inputs, four hidden neurons with ReLU activation, and one output neuron, was estimated to be less than 1 millisecond per cycle, representing only 0.025% of the total sampling period. This time includes inference, error, and integral calculation, as well as PWM signal generation operations. Considering that the HC-SR04 ultrasonic sensor reading requires approximately 25 ms, the total detection, control, and actuation cycle time remains well below 1% of the sampling interval, ensuring real-time execution without noticeable delays or degradation of system stability. Table 6 summarizes the estimated values to quantify the temporal performance of the implemented controller.

Finally, the hyperparameters considered correspond only to the DDPG agent training process, while the physical and simulation parameters of the plant were defined independently in Section 2.1 and Section 2.3. On the other hand, the size of the neural networks was determined empirically. Likewise, the selected architecture consists of 50–25 neurons for the critical network and 4 for the actor network.

## 3. Results and Discussion

This section presents the main results obtained after training and implementing the controller based on the DDPG algorithm and applied to the water level control system. To ensure realistic behavior of the simulation environment, the physical parameters of the tank obtained through experimental tests were used, which are summarized in Table 7.

The DDPG agent was trained on a laptop with a 3.20 GHz AMD Ryzen 7 6800HS processor, 8 GB of RAM, and a 64-bit Windows 10 Pro operating system, version 22H2. MATLAB R2020b software was used with the Reinforcement Learning Toolbox to develop the simulation environment, train the agent, and analyze the results. The simulation was built from the dynamically obtained model, which was used to represent the behavior of the real system during the reinforcement learning process.

The physical implementation was carried out using an Arduino Uno R3 microcontroller, which has an ATmega328P microcontroller, (Manufacturer: Microchip Technology Inc.; Chandler, AZ, USA) 2 KB of RAM, 32 KB of flash memory, and a 10-bit analog-to-digital converter (ADC). The acquisition and control system incorporated an HC-SR04 ultrasonic sensor for measuring the water level, a 9 V pump as an actuator, and an L298N driver module for power control. Communication between MATLAB and the microcontroller was carried out via a serial port using the USB interface, allowing real-time application of the trained policy.

### 3.1. Simulation Results

**Figure 10:**
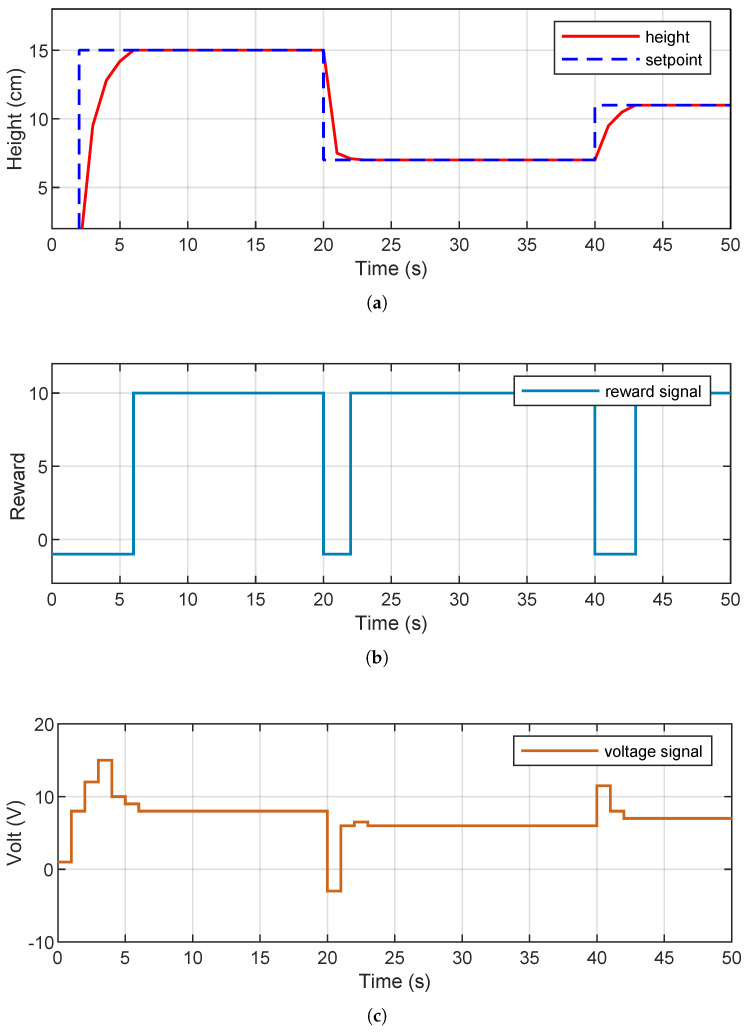
System evolution. (**a**) Height versus setpoint. (**b**) Reward. (**c**) Applied voltage.

Figure 10 presents the results obtained during the simulation. Figure 10a shows the system’s response to different reference values (setpoints). It can be seen that the agent manages to follow these references accurately, with smooth transitions and without overshoots, which demonstrates the trained controller’s adequate generalization capacity. Figure 10b shows the evolution of the accumulated reward throughout the training. The sustained upward trend indicates a progressive improvement in the learned policy, reflecting an adequate configuration of both the reward function and the model’s hyperparameters. Finally, Figure 10c shows the PWM signal generated by the agent. It presents smooth and adaptive variations, demonstrating an efficient response to changes in reference levels.

Table 8 shows the performance metrics obtained in the simulation. These metrics, calculated from the time response, allow for a quantitative evaluation of the controller’s performance in the simulation. Likewise, the metrics reflect satisfactory controller performance; i.e., the response is fast and stable, without unwanted oscillations, and with low steady-state error. These results confirm the effectiveness of the DDPG-trained agent in the simulated environment and support its subsequent implementation in the physically embedded system.

To establish a comparative reference, a PID controller tuned using the Ziegler–Nichols method was simulated under the same plant conditions. The PID controller achieved a steady-state error of 1.4 cm and a rise time of 3.8 s, compared to 0.84 cm and 2.2 s achieved by the DDPG agent. This represents a 40% improvement in accuracy and a 42% faster response, confirming the advantage of deep reinforcement learning in nonlinear environments.

### 3.2. Physical Implementation Results

This section presents the experimental results obtained when implementing the DDPG controller in an embedded environment based on an Arduino Uno microcontroller, replicating the conditions used in the simulation.

**Figure 11:**
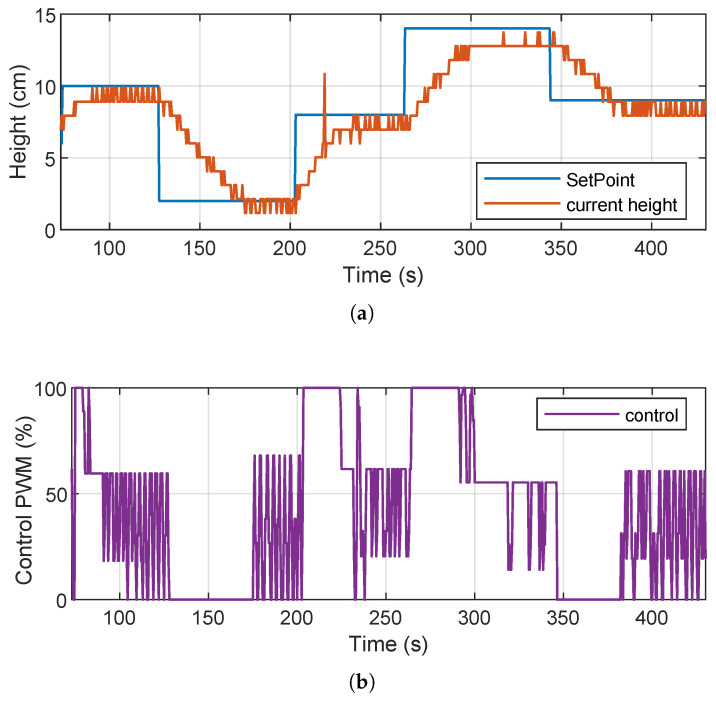
Results of the physical implementation: system response to different references. Bottom: PWM control signal generated by the DDPG controller. (**a**) Response to different references. (**b**) PWM control signal.

Figure 11 shows the results obtained during testing on the real system. The upper part shows the evolution of the water level as a function of the different references or setpoints. The lower part shows the PWM control signal generated by the DDPG agent running in the embedded environment. On the other hand, we can see in Figure 11a that the physical system manages to reach the different setpoints imposed, generally validating the effectiveness of the trained controller. However, we can also observe some irregularities in the measured water level signal, such as consecutive peaks once the level has stabilized and step-like or *staircase* transitions. These behaviors are attributed to noise and instability in the ultrasonic sensor readings, which introduce uncertainty in the measurement and affect the smoothness of the response. On the other hand, as shown in Figure 11b, the PWM control exhibits suboptimal behavior; i.e., it repeatedly produces abrupt oscillations between distant values instead of generating smooth and continuous variations. This pattern suggests that the agent may be overreacting to sensor fluctuations, attempting to stabilize the level through drastic changes that are neither energy efficient nor desirable for the actuator. It is also possible that the agent is averaging these oscillations to keep the level within the set point, which coincides with the small zigzags observed in the level response.

Despite these limitations, the controller shows a basic regulation capacity, achieving the reference targets with a low steady-state error. Table 9 shows the classic time response metrics obtained in the implementation of the system. These metrics show that while the controller achieves the desired values, its performance can be affected by physical environment factors, such as sensor noise and the limited processing capacity of the embedded hardware. In addition, opportunities for improvement are identified in both signal filtering and the agent’s robustness to noisy measurements. Overall, the results confirm the initial feasibility of implementing a DDPG agent in a low-cost embedded environment. However, they also highlight the importance of making adjustments to the sensory integration and controller design to achieve more efficient and stable behavior in real-world applications.

### 3.3. DDPG Controller Performance Analysis

The performance of the DDPG controller was evaluated both in simulation and physical implementation, considering metrics such as rise time, settling time, overshoot, undershoot, and steady-state error.

In the simulation, the controller showed a fast and stable response, with a settling time of less than 6 s and no overshoot or undershoot, achieving accurate setpoint tracking. In addition, the control signal generated was smooth and continuous, reflecting efficient action by the agent in response to reference changes. In the physical implementation, although the system was able to adequately follow the different setpoints, several limitations inherent to the real environment were evident. The response time was considerably longer due to hardware latency and noise inherent in the ultrasonic sensor measurements. This noise generated oscillations in the signal, especially once the setpoint was reached, causing small fluctuations in the system response that were not observed in the simulation. Likewise, the PWM control signal generated by the agent exhibited erratic behavior, alternating significantly between high and low values, even in a steady state. This pattern suggests that the agent could compensate for sensor noise with extreme actions, which affects control efficiency and could cause greater wear on the actuator in the long term. This behavior also contrasts with what was observed in the simulation, where the PWM signal was smoother and more gradual.

Finally, the experimental adjustment of hyperparameters was performed experimentally through exploratory testing. During the adjustment, it was observed that significant variations in the learning rate directly affected stability and convergence speed. High learning rates generated oscillations in the reward, while values that were too low slowed down the learning process. Minibatch sizes greater than 128 did not offer notable improvements. Intermediate minibatch sizes provided the best balance between performance and stability. Minibatch sizes smaller than 32 reduced stability. On the other hand, in relation to the size of the neural networks, the proposed architecture (50–25 neurons for the critical network and 4 for the actor network) offered the best balance between representation capacity, stability, and computational feasibility for its embedded implementation, since a few neurons caused erratic rewards. In contrast, an excessive number of neurons did not improve performance. Thus, the configuration in the architecture ensured stable learning and consistent controller performance.

**Figure 12:**
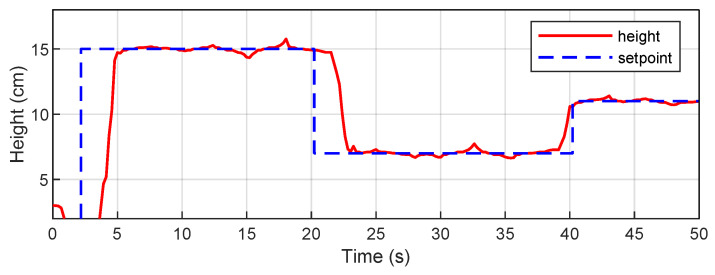
DDPG controller simulation in the presence of sensor noise and actuator delay.

**Figure 13:**
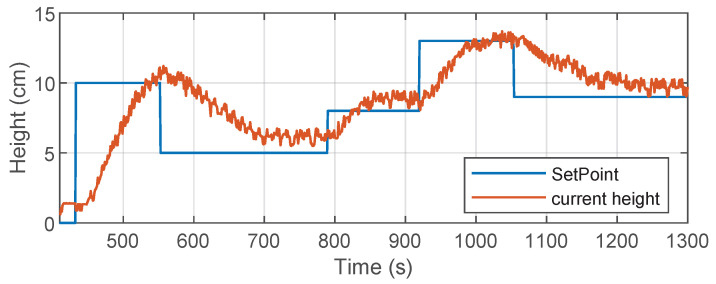
Performance of a PID controller, tuned using the Ziegler–Nichols method.

Additionally, to verify the experimental validity of the study, the behavior of the proposed system was evaluated under non-ideal conditions and compared with a classic PID controller. Figure 12 shows the results of the DDPG controller simulation in the presence of sensor noise and actuator delay. The agent maintains stable tracking under these disturbances and retains a smooth response, albeit with small oscillations induced by measurement noise. This confirms the robustness of the controller when operating under non-ideal conditions. Figure 13 shows the performance of a PID controller, tuned using the Ziegler–Nichols method, in the actual physical system. The system reaches the reference levels with an overshoot of approximately 15% and has a longer stabilization time compared to the DDPG controller. More pronounced oscillations can also be observed. These results provide a valuable benchmark for contrasting the performance of the reinforcement learning-based controller.

### 3.4. Comparison with Previous Studies

The results obtained in this work are consistent with previous research on the development and application of the DDPG algorithm in various control contexts. Table 10 summarizes the most relevant studies that have explored DDPG-based control strategies, highlighting their validation methods and main results.

Several studies have focused on simulated implementations. For example, Kuo et al. [31] proposed a wave-based DDPG controller (W-DDPG) combined with sequential sensor fusion for bipedal locomotion, achieving stable and adaptive gait generation with greater robustness against external disturbances. However, since their experiments were conducted entirely in simulation, they did not address hardware-specific issues such as actuator latency, sensor noise, or nonlinear dynamics. Similarly, Syafiie et al. [21] applied DDPG for multivariable level and pH control, demonstrating greater accuracy compared to conventional PID controllers. However, their evaluation was also limited to a simulated environment. On the other hand, Zhao et al. [15] implemented a DDPG-based controller for HVAC regulation, achieving lower energy consumption while maintaining thermal comfort. Building on this line of work, Zhang and Lam [20] carried out one of the few real-world implementations of a DRL-based controller, applying it to a radiant heating system in an actual office building. Their results confirmed the feasibility of DRL control in physical environments, but also highlighted challenges such as model calibration, data requirements, and computational limitations. In contrast, the present study is distinguished by performing both the simulation and the complete hardware implementation of a DDPG-based controller, experimentally validating the learned policy in a physical system. This two-stage validation demonstrates the effectiveness of the algorithm under real-world conditions, including sensor noise and actuation delays.

#### Comparison with Studies on Fuzzy PID or MPC in Water Tank Systems

Table 11 presents a comparison of the results obtained in this study with those reported in recent research using conventional control strategies, such as Fuzzy-PID and MPC. In the work of Saad et al. [32], the Fuzzy-PID controller significantly reduced the settling time and eliminated overshoot compared to the classic PID. Similarly, Montaluisa et al. [33] demonstrated that MPC improved response times compared to PI, albeit with greater computational complexity. Finally, Kassie et al. [34] showed that GA-PID offered shorter rise times than Fuzzy-PID in a two-tank system. In comparison, the proposed DDPG controller achieves a settling time of approximately 74.45 s and a steady-state error of less than 1 cm in physical implementation, demonstrating performance improvement over traditional methods. Furthermore, this work shows an additional advantage of autonomous learning and viability in low-cost embedded hardware.

The impact of the results of this study is oriented towards two contexts. Firstly, in the industrial context, the results demonstrate the feasibility of implementing DRL-based controllers on low-cost integrated platforms, opening up the possibility of developing intelligent control systems for hydraulic, agricultural, or energy processes without relying on specialized hardware. The combination of an Arduino microcontroller and a pre-trained DDPG agent provides an economical, scalable, and adaptable solution for process automation in small- and medium-sized industries, thus contributing to energy efficiency and sustainable water resource management. Secondly, in the academic context, the results constitute an experimental validation of the integration between DL techniques and real-time control, strengthening the bridge between simulation and practical application. The proposed framework, which is open source and reproducible, can serve as a reference for future research in intelligent control, as well as for teaching reinforcement learning in engineering and applied science courses. Taken together, the results contribute to consolidating the use of deep reinforcement learning in real embedded systems and encourage new lines of research in adaptive and intelligent control.

Finally, the results correspond to representative tests, given that repeated experiments showed qualitatively similar responses. However, insufficient quantitative measurements were recorded to calculate descriptive statistics (mean ± standard deviation). This aspect represents a limitation of this research, which may be addressed in future work.

## 4. Conclusions

This work presented the design, training, and implementation of an intelligent controller based on DDPG for regulating tank water levels. The proposed agent was trained in a simulated environment, achieving a precise and stable control policy. This policy was then implemented in an embedded system using an Arduino Uno microcontroller and an HC-SR04 ultrasonic sensor. Furthermore, comparison with a classic PID controller showed that the DDPG agent achieved smoother responses and reduced oscillations under non-ideal conditions, highlighting its superior robustness. Hyperparameter tuning was performed experimentally to optimize the learning rate, minibatch size, smoothing coefficients, and exploration noise. This process stabilized training and improved convergence. The cumulative reward increased by up to 85%, indicating a progressive improvement in the learned policy. In summary, unlike previous studies focused on simulation, this work provides experimental evidence on the feasibility and performance of deploying DDPG agents in real embedded systems, representing an important step towards their adoption in industrial and low-cost applications. Furthermore, the comparative analysis with recent Fuzzy-PID, GA-PID, and MPC studies shows that the proposed DDPG controller offers competitive settling times and significantly lower steady-state errors, while also providing autonomous learning capabilities not present in conventional methods. In summary, while traditional controllers rely on fixed-gain tuning, the DDPG approach allows for dynamic adaptation to nonlinearities and unmodeled disturbances. The agent’s generalization ability suggests potential applications in temperature control, flow control, or smart agricultural processes. These results reinforce the feasibility of scaling deep reinforcement learning to low-cost microcontroller platforms.

## 5. Future Work

This work opens up new lines for future research, such as analyzing agent performance optimization using more advanced techniques for automatic hyperparameter tuning, incorporating online adaptive learning mechanisms, and conducting experimental comparisons with traditional controllers, such as PID or fuzzy controllers. Another line for future research is to consider extending the approach to multivariable systems or systems with more complex physical constraints. Although the experimental results presented in this work confirm the viability and effectiveness of the proposed DDPG-based control strategy, a direct comparison with traditional controllers such as PID or fuzzy logic is not included. Such a comparative analysis would provide valuable benchmarks for evaluating improvements in performance, robustness, and computational efficiency. Also, research should integrate real-time data streaming and online learning through edge and fog computing architectures, enabling adaptive retraining in response to changing environmental conditions. Furthermore, a comparative study with PID and fuzzy controllers will provide quantitative benchmarks for performance and energy consumption. Finally, future work may address the performance of multiple repetitions to estimate the experimental variability of the proposed controller. The objective of this implementation is to record quantitative measurements and determine descriptive statistics, such as the mean ± standard deviation. In addition, future improvements may address the small oscillations observed in the PWM signal during implementation. To mitigate this effect, a first-order digital smoothing filter may be applied to the actor output, acting as a low-pass filter that attenuates rapid variations without significantly affecting the system dynamics. Alternatively, a maximum step-change constraint between consecutive control cycles may be implemented to limit abrupt variations in the PWM signal.

## Figures and Tables

**Table 1:** General notation.

Description	Notation	Equation
Inflow	$Q_{e}$	(1)
Outflow	$Q_{s}$	(2)
Cross-sectional area of the tank	*A*	
Water height in the tank	*H*	
Acceleration due to gravity	*g*	
Outlet valve gain	$k_{2}$	(6)
Motor gain	$k_{1}$	(5)
Average motor voltage	$u_{1}$	
Outlet valve opening degree	$a_{2}$	
Observation state	o(t)	(10)
Action state	a(t)	(11)
Reward state	R(t)	(12)
Critical target value	$y_{i}$	(7)
Actor policy gradient	$\nabla_{\theta^{\mu }}J$	(8)
Smooth update of target networks	$\theta^{Q^{\prime }},\theta^{\mu^{\prime }}$	(9)

**Table 2:** Estimation of $k_{1}$ and $k_{2}$.

Parameter	Estimated Value	Unit
$k_{1}$	$9\times 10^{-6}$	[m/s]
$k_{2}$	$6.87\times 10^{-4}$	$\left[m^{1/2}/s\right]$

**Table 3:** Critical network architecture.

Subnet	Layer	Neurons	Activation
State	input	3	–
State	FC1	50	ReLU
State	FC2	25	–
Action	input	1	ReLU
Action	FC1	25	–
Common	sum + ReLU	–	ReLU
Common	FC	1	Linear

**Table 4:** Actor network architecture.

Layer	Neurons	Activation
input	3	–
1	4	ReLU
2	1	Linear

**Table 5:** Parameters used in the DDPG controller.

Description	Parameter	Value
Actor learning rate		$1\times 10^{-4}$
Critic learning rate		$1\times 10^{-3}$
Discount rate	γ	1.0
Repeat buffer size	R	$1\times 10^{6}$
Minibatch size	*D*	64
Soft update parameter	τ	$1\times 10^{-3}$
Scan noise		0.3
Noise decay		$1\times 10^{-5}$
Number of episodes		$1\times 10^{3}$
Sampling time	$T_{s}$	1.0
Steps per episode	$T_{f}$	200

**Table 6:** Estimated execution times for each stage of the control cycle.

Control Cycle Stage	Function	Estimated Time
Reading of the HC-SR04 sensor	Pulse generation and reception	≈25 ms
Calculation of error and integral	Simple arithmetic operations	<1 ms
Inference of the actor neural network (ReLU)	Multiplications and additions	≈25 ms
PWM update of the motor pump	Analogue writing	≈0.1 ms
Total loop delay	Calculation of the total sum	≈26 ms
Sampling period	Control executed every 4 s	4000 ms

**Table 7:** Physical parameters of the water tank.

Parameter	Value
*A*	0.02269 m^2^
$H_{max}$	0.185 m
$k_{1}$	$9\times 10^{-6}$
$k_{2}$	$6.87\times 10^{-4}$
$a_{2}$	50%
*g*	9.8 m/s^2^

**Table 8:** Performance metrics obtained in simulation.

Metric	Value
Rise time	2.20 s
Settling time	5.15 s
Maximum value	15.06 cm
Time to peak	50.00 s
Overshoot	0.00 cm
Undershoot	0.00 cm
Steady state error	0.84 cm

**Table 9:** Performance metrics obtained from hardware implementation.

Metric	Value
Rise time	18.50 s
Settling time	74.45 s
Maximum value	6.96 cm
Time to peak	31.00 s
Overshoot	16.00 %
Undershoot	0.00 cm
Steady state error	0.035 cm

**Table 10:** Comparison of relevant studies applying DDPG-based control strategies.

Ref.	Controlled System	Validation of Tests	Platform	Key Results
[31]	Biped robot	Simulation	Simulated environment	Stable and adaptive gait generation using W-DDPG with sensor fusion
[21]	Multivariable process (level and pH)	Simulation	Process simulator	Higher precision than conventional PID controllers
[15]	HVAC system	Simulation	Multivariable environment	Reduced energy consumption while maintaining thermal comfort
[20]	Radiant heating system	Real deployment	Building testbed	Energy savings achieved; highlighted calibration and computational challenges
**Our study**	Water level control system	Full experimental	Arduino Uno + ultrasonic sensor	Error < 5%; stable control under noise and disturbances

**Table 11:** Comparison of relevant studies on Fuzzy PID or MPC in water tank systems.

Ref.	Controller	Validation of Tests	Rise Time (s)	Settling Time (s)	Overshoot (%)
[32]	PID/Fuzzy–PID	Simulation	8.45	23.28	12
[33]	PI/MPC	Experimental without disturbance	622.5	745	0.9
[34]	Fuzzy-PID/GA-PID	Simulation	0.039	8.50	−0.39
**Our study**	DDPG	Physical implementation	18.50	74.45	16

## Data Availability

The public repository with the source code and files needed to reproduce this work is available at: https://github.com/kevin37773/Control-nivel-de-agua-con-aprendizaje-reforzado-profundo-DDPG- (accessed on 1 September 2025).
